# Pearl Syndrome With an Unusual Association of Spina Bifida and Congenital Cholesteatoma

**DOI:** 10.7759/cureus.22832

**Published:** 2022-03-04

**Authors:** Pratiksha S Nathani, Revathy Krishna, Vikas Solunke, Shivprasad Mundada

**Affiliations:** 1 Pediatrics, Vilasrao Deshmukh Government Medical College, Latur, IND

**Keywords:** pearl, congenital cholesteatoma, spina bifida, congenital heart disease, microtia, seventh cranial nerve palsy, congenital facial nerve palsy

## Abstract

We report a case of a one-day-old female with congenital facial nerve palsy, bilateral microtia, congenital heart disease, spina bifida, and congenital cholesteatoma. The newborn was brought by the mother with complaints of abnormally looking ear and a facial droop toward the left side, following which a two-dimensional echocardiography was done showing the atrial septal defect and ventricular septal defect. Computed tomography of the temporal bone showed the presence of congenital cholesteatoma in the left ear. MRI of the lumbosacral spine was suggestive of spina bifida occulta. Brainstem evoked response audiometry was suggestive of sensorineural hearing loss. Such a combination of symptoms is very rare, and therefore this case is being reported.

## Introduction

Pearl syndrome has a triad of congenital facial palsy, anotia, and congenital heart disease [1]. It is often discussed in the literature whether this triad is a variant of Goldenhar syndrome [2]. Facial nerve palsy is a common congenital defect presenting as a part of a syndrome or separately. It is usually idiopathic and resolves completely in the majority of cases, but in some cases, it may have residual weakness or contracture, and sometimes hemifacial spasm [3]. Furthermore, facial palsy also has psychological impacts like anxiety and distress [4]. Microtia can present as hearing loss and can occur as an isolated presentation or it can present as a genetic syndrome due to a single abnormal gene [5]. Congenital heart defects presenting as shortness of breath or cyanosis require prompt diagnosis and intervention and can lead to complications like development delays, rhythm abnormalities, stroke, and mental health issues [6]. In this reported case, microtia served as a diagnostic clue for the association with a syndrome and was followed by a thorough examination and investigations, which led to findings of a rare cluster of bilateral microtia, congenital facial palsy, congenital heart disease, spina bifida, and congenital cholesteatoma.

## Case presentation

**Figure 1:**
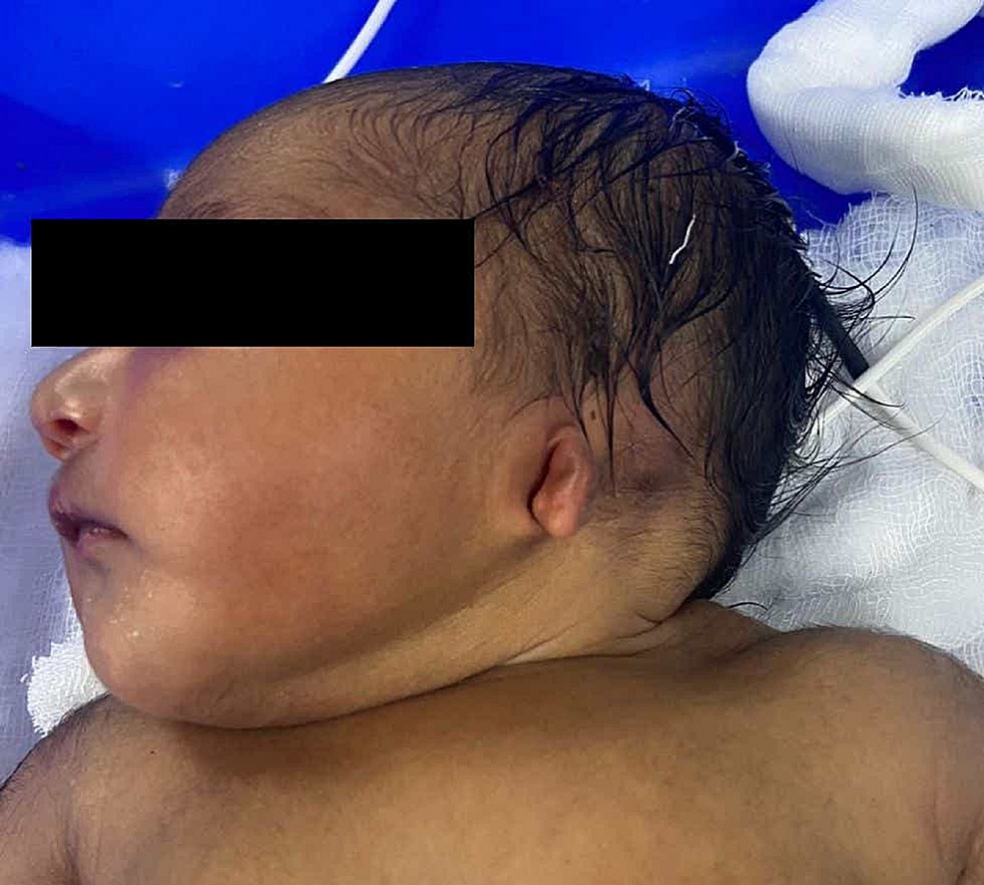
Microtia on the left ear.

**Figure 2:**
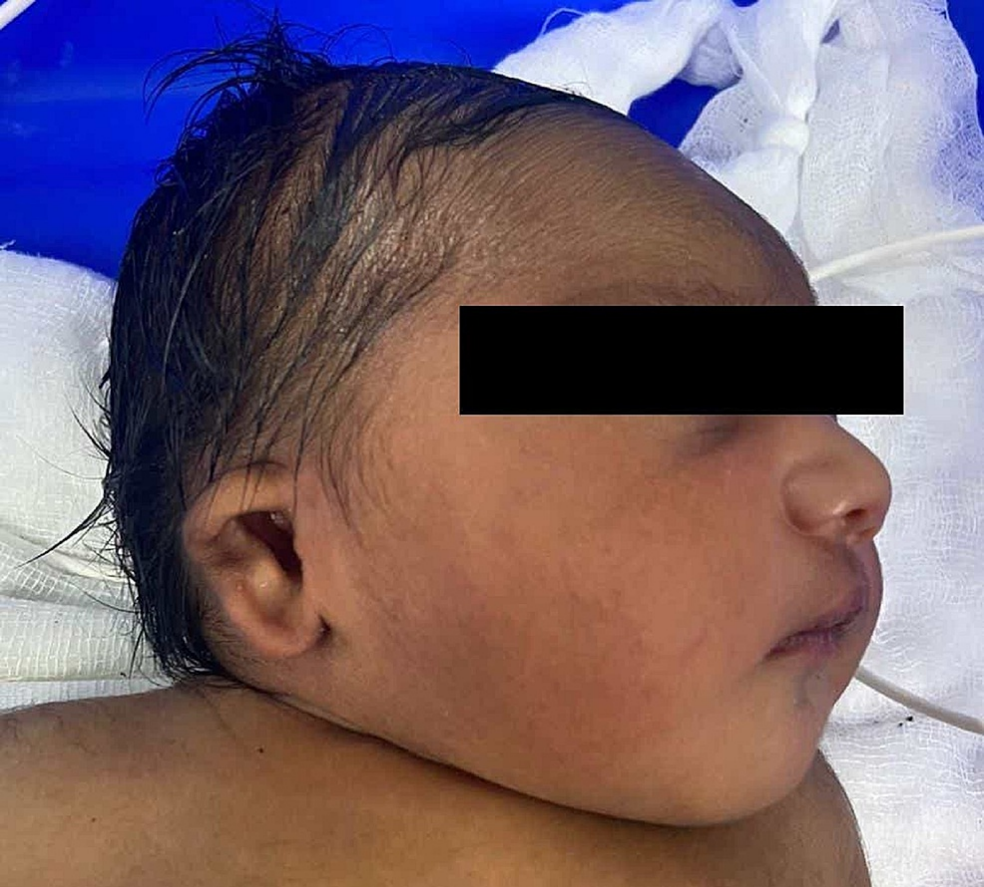
Microtia on the right ear.

**Figure 3:**
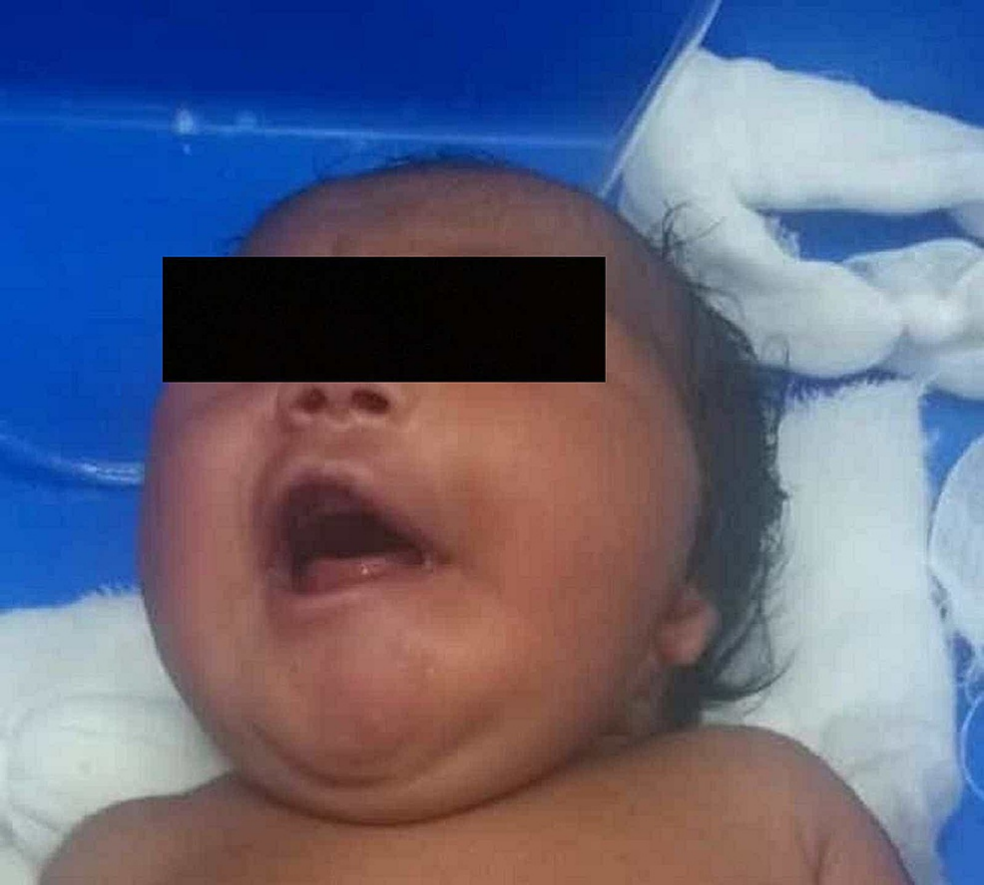
Facial droop on the left side while crying and absent nasolabial fold on the left side.

**Figure 4:**
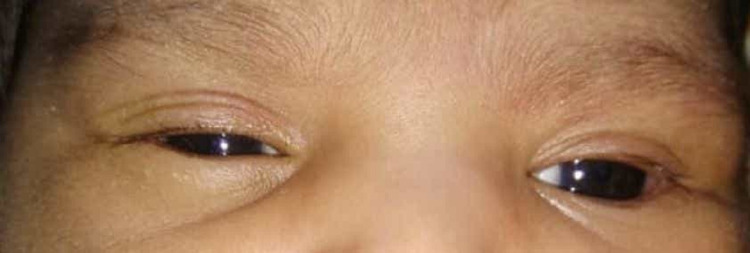
Inability to close the left eye suggesting left-sided weakness.

A fresh newborn term female was admitted to the neonatal intensive care unit of Vilasrao Deshmukh Government Medical College, Latur, India in view of both deformed ears. She was a first-order term, adequate for gestational age, born from a nonconsanguineous marriage, from normal vaginal delivery to a 20-year-old primigravida. The baby cried immediately after birth. There was no history of fever, hypertension, gestational diabetes, intake of any teratogenic drugs, or exposure to any radiation in the antenatal period. Iron, calcium, and folic acid supplements along with two doses of tetanus toxoid were taken by the mother. At birth, the recorded birth weight was 3.1 kg, length was 49 cm, and head circumference was 32 cm with right and left-sided microtia, as can be seen in Figures 1, 2. She presented facial droop toward the left while crying and absence of the left nasolabial fold, as seen in Figure 3. The inability to tightly close the left eyelid was evident, suggesting congenital facial nerve palsy, which was later confirmed by the seventh cranial nerve conduction test, as seen in Figure 4.

On examination, the general condition was good, with a heart rate of 146 beats per minute and a respiratory rate of 56 breaths per minute. Chest bilateral air entry was equal and normal first heart sound and soft second heart sound with pansystolic murmur over the complete precordium were noted. External examination of the spine did not show any presacral dimple or tuft of hairs. No manifestations of any other cranial nerve palsy were present, and the rest of the nervous system examination was normal.

**Figure 5:**
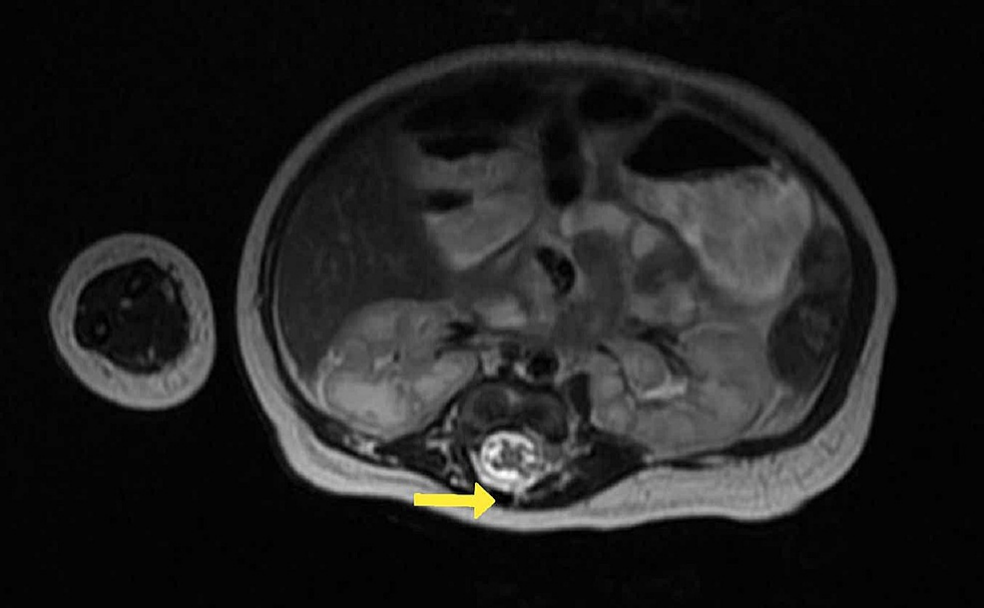
Axial T2 MRI image showing absent transverse process with overlying skin.

**Figure 6:**
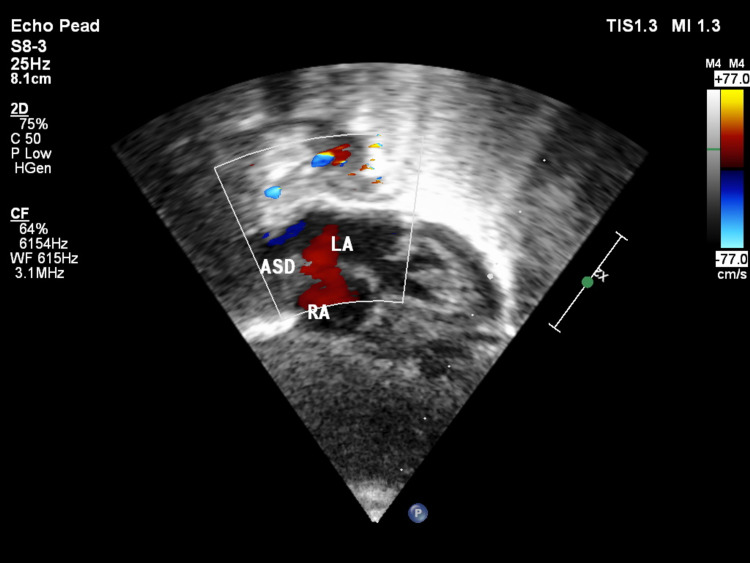
Two-dimensional echocardiogram image showing the atrial septal defect.

**Figure 7:**
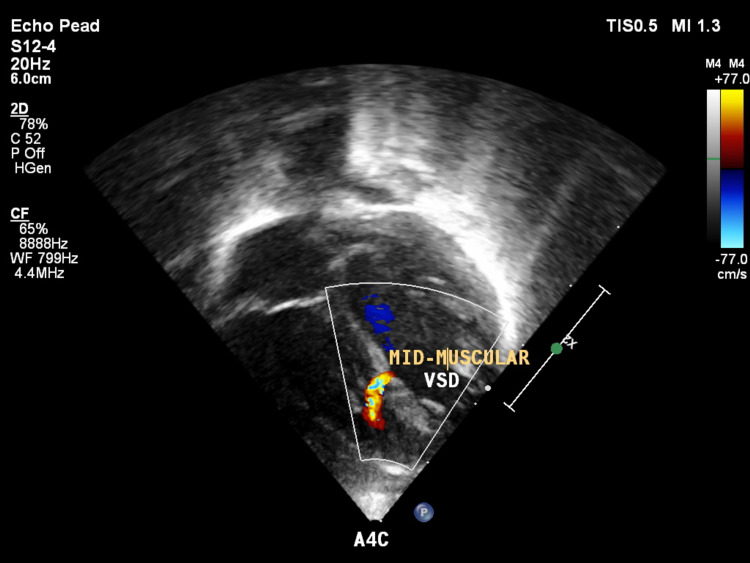
Two-dimensional echocardiogram image showing the mid-muscular ventricular septal defect.

USG local showed a 1-cm-sized defect in the posterior elements of vertebrae at the thoracolumbar region without herniation of meninges and spinal cord, which was suggestive of spina bifida. Antenatal USG also showed evidence of spina bifida. USG was followed up with an MRI of the lumbosacral spine, which showed hypoplastic deformed splaying of posterior elements of dorsosacral vertebrae resulting in focal dilation of the vertebral canal without herniation of neural elements suggestive of spina bifida occulta, as seen in Figure 5. Two-dimensional echocardiography was suggestive of moderate secundum atrial septal defect and small mid-muscular ventricular septal defect, as seen in Figures 6, 7.

Computed tomography of the temporal bone was suggestive of left-sided congenital cholesteatoma, dysplastic middle ear ossicles, hypoplastic left ear auricle, and external auditory canal. This was followed by brainstem evoked auditory response, which confirmed severe sensorineural hearing loss in both ears.

Total body X-ray and a cervicothoracic vertebral column X-ray revealed dysplastic thoracolumbar vertebra with partial fusion of anterior and posterior elements. Ocular examination was not suggestive of any abnormalities and hence ruled out Goldenhar syndrome. MRI of the brain and USG of the abdomen were normal.

Pathological complete blood count, serum direct total bilirubin, serum electrolytes, and serum calcium levels were normal.

## Discussion

The maximum number of cases to be reported with microtia, congenital heart disease, and central nervous system abnormalities are with isotretinoin as retinoic acid embryopathy, Goldenhar syndrome, thalidomide embryopathy, fetal alcohol syndrome, 22q11 deletion syndrome, and use of antiepileptic drugs during pregnancy, which are well documented in the literature. A rare syndrome was reported by Pearl in 1984 with a triad of anotia, facial paralysis, and congenital heart disease without any evident cause [1]. Later, this syndrome has been associated with other syndromes like Jacobs syndrome [7]. Microtia is a congenital deformity of the ear in which the ears are smaller than the normal size to a severe form, anotia, where there is a complete absence of the ear mostly leading to hearing loss. The ears are usually malformed in such cases. Causes of congenital microtia are maternal type 1 diabetes mellitus, intake of alcohol, and specific drugs like isotretinoin, thalidomide, and mycophenolate mofetil [8]. No such cause was reported in this case.

Congenital facial palsy can be a part of a syndrome or can occur as a solitary anomaly. When it occurs congenitally, the prognosis is usually not good [9]. Prenatal factors play a role in congenital facial palsy. Instrumentation during delivery is considered a significant factor for traumatic facial palsy but has a spontaneous recovery within a few months [10]. There was no history of any instrumentation during delivery in this case. It can be associated with abnormalities like microtia, atresia, hemifacial microsomia, facial clefts, Moebius syndrome, and congenital conductive sensorineural loss [11]. This reported case has a conductive sensorineural loss. Conductive component is due to the presence of congenital cholesteatoma as seen on MRI and sensorineural loss as confirmed on brainstem evoked auditory response.

Congenital heart diseases contribute a significant amount of burden to health care. A cohort study conducted in India concluded that congenital heart diseases were the most common congenital anomaly in India followed by neural tube defects [12]. This case has both a congenital heart defect and a neural malformation. About 30% of the congenital heart morbidity is a part of genetic syndromes like Down syndrome, Turner syndrome, and 22q11 deletion syndrome [13]. Ventricular septal defect and atrial septal defects of less than 5 mm should close by themselves within the first year of life as seen in the majority of cases [14,15]. Medications like valproic acid during the initial months of pregnancy, smoking, hyperglycemia due to gestational diabetes mellitus, and deficiency of folic acid in mothers are some established risk factors for neural tube defects in newborns [16]. No such risk factor was reported by the mother, and folic acid supplements were taken by her during the pregnancy period.

## Conclusions

The case shows the incidence of a very rare syndrome having the presence of congenital facial palsy, bilateral microtia, congenital heart disease superimposed with spina bifida, and congenital cholesteatoma as a very uncommon association. Since no speciﬁc risk factor or causative agent was found in this case, it was concluded to be of unknown etiology. When contemplated, this unknown syndrome can be said as Pearl syndrome with an unusual association of spina bifida and congenital cholesteatoma. Microtia and congenital facial palsy can be used as significant clues while diagnosing congenital syndromes as in this case. This case also highlights the need for screening of every high-risk newborn for highly prevalent congenital defects, and when found, they should be correlated with relevant syndromic associations so that early diagnosis and efficient line of treatment options can be set up for a good prognosis and improved quality of life in such cases.
